# Deregulation of polycomb repressor complex 1 modifier AUTS2 in T-cell leukemia

**DOI:** 10.18632/oncotarget.9982

**Published:** 2016-06-13

**Authors:** Stefan Nagel, Claudia Pommerenke, Corinna Meyer, Maren Kaufmann, Hans G. Drexler, Roderick A.F. MacLeod

**Affiliations:** ^1^ Department of Human and Animal Cell Lines, Leibniz-Institute DSMZ – German Collection of Microorganisms and Cell Cultures, Braunschweig, Germany

**Keywords:** IL-7, IL7R, MEF2C, PRC1, PRC2

## Abstract

Recently, we identified deregulated expression of the B-cell specific transcription factor MEF2C in T-cell acute lymphoid leukemia (T-ALL). Here, we performed sequence analysis of a regulatory upstream section of MEF2C in T-ALL cell lines which, however, proved devoid of mutations. Unexpectedly, we found strong conservation between the regulatory upstream region of MEF2C (located at chromosomal band 5q14) and an intergenic stretch at 7q11 located between STAG3L4 and AUTS2, covering nearly 20 kb. While the non-coding gene STAG3L4 was inconspicuously expressed, AUTS2 was aberrantly upregulated in 6% of T-ALL patients (public dataset GSE42038) and in 3/24 T-ALL cell lines, two of which represented very immature differentiation stages. AUTS2 expression was higher in normal B-cells than in T-cells, indicating lineage-specific activity in lymphopoiesis. While excluding chromosomal aberrations, examinations of AUTS2 transcriptional regulation in T-ALL cells revealed activation by IL7-IL7R-STAT5-signalling and MEF2C. AUTS2 protein has been shown to interact with polycomb repressor complex 1 subtype 5 (PRC1.5), transforming this particular complex into an activator. Accordingly, expression profiling and functional analyses demonstrated that AUTS2 activated while PCGF5 repressed transcription of NKL homeobox gene MSX1 in T-ALL cells. Forced expression and pharmacological inhibition of EZH2 in addition to H3K27me3 analysis indicated that PRC2 repressed MSX1 as well. Taken together, we found that AUTS2 and MEF2C, despite lying on different chromosomes, share strikingly similar regulatory upstream regions and aberrant expression in T-ALL subsets. Our data implicate chromatin complexes PRC1/AUTS2 and PRC2 in a gene network in T-ALL regulating early lymphoid differentiation.

## INTRODUCTION

In T-cell acute lymphoid leukemia (T-ALL) several types of oncogenes are involved in leukemogenesis deregulating survival, proliferation and developmental processes, resulting in differentiation arrest of developing T-cells at particular stages [1–3]. T-cell differentiation is mainly regulated at the transcriptional level creating gene regulatory networks [4]. Therefore, deregulation of transcription factors (TFs) represents a basic mechanism of T-cell transformation. Homeobox genes encode particular TFs which perform fundamental functions in developmental processes. Several genes of the NKL subclass are aberrantly activated in T-ALL and deregulate cellular differentiation of thymocytes. The chromosomal rearrangement t(10;14)(q24;q11) activates NKL homeobox gene TLX1, and t(5;14)(q35;q32) activates TLX3 or NKX2-5 [5–8]. Aberrant activation of NKL homeobox gene MSX1 is not mediated by chromosomal alterations but via deregulation of the BMP-signalling pathway [9]. This gene is physiologically expressed in early lymphopoiesis and silenced during ensuing differentiation steps [10]. Accordingly, MSX1 represses differentiation of other tissues/cell types as well, including muscle cells and cardiomyocytes [11, 12]. Therefore, repression of differentiation may represent a common outcome of aberrantly expressed structurally and functionally related NKL homeobox genes for thymocyte transformation [9].

Polycomb repressor complex (PRC)1 and PRC2 represent transcriptional repressors of developmental genes, including homeobox genes [13–15]. These complexes consist of several proteins forming subtypes according to composition, localization and function [15–17]. Aberrant downregulation of PRC2 member EZH2 has been reported in T-ALL resulting in activation of the clustered homeobox gene HOXA10 [18]. Furthermore, in T-ALL and early T-cell progenitor (ETP)-ALL inactivating mutations have been found in EZH2 and additionally in EED and SUZ12 [19–21]. In contrast, in adult T-cell leukemia the activity of PRC2 is aberrantly enhanced [22], highlighting the importance of a balanced activity of regulatory chromatin complexes for normal lymphoid development.

MEF2C encodes a MADS-box TF which regulates the development, proliferation and survival of B-cells, and is silenced in T-cells [23–25]. Aberrant activation of MEF2C during T-cell differentiation contributes to leukemogenesis in a subset of T-ALL associated with an immature phenotype [26, 27]. In this malignant context MEF2C transcription is directly activated by the oncogenic TF NKX2-5 or via chromosomal aberrations including T-cell receptor gene translocation or upstream deletion. The genomic deletion of its regulatory upstream region removes a repressive STAT5 binding site thus promoting aberrant activation [26, 28].

The advent of next generation sequencing and the analyses of whole cancer genomes have revealed oncogenic mutations located in protein coding genes in addition to regulatory gene regions targeting particular TFBS. Recently, a mutated binding site for GATA3 in a super-enhancer of the LMO1 gene has been identified in neuroblastoma, and the mutagenic formation of a TFBS for TCFL2 in the MYC gene mediates elevated expression in intestinal tumors [29, 30]. Thus, TFBS mutations represent a basic but probably underestimated mechanism of oncogenic aberration.

The foregoing considerations prompted our enquiry into the wider role of TFBS mutations in T-ALL as well. Therefore, we sequenced in T-ALL cell lines the reported STAT5 binding site at MEF2C which mediates transcriptional repression via IL7 signalling [28]. However, while failing to detect TFBS mutations thereat, this approach uncovered striking regulatory sequence conservation between MEF2C at chromosomal band 5q14 and AUTS2 at 7q11. Subsequent investigations revealed novel roles for AUTS2 as chromatin modulator impacting early lymphoid development and T-cell leukemogenesis.

## RESULTS

### Concurrent regulatory sequences at MEF2C and AUTS2

**Figure 1:**
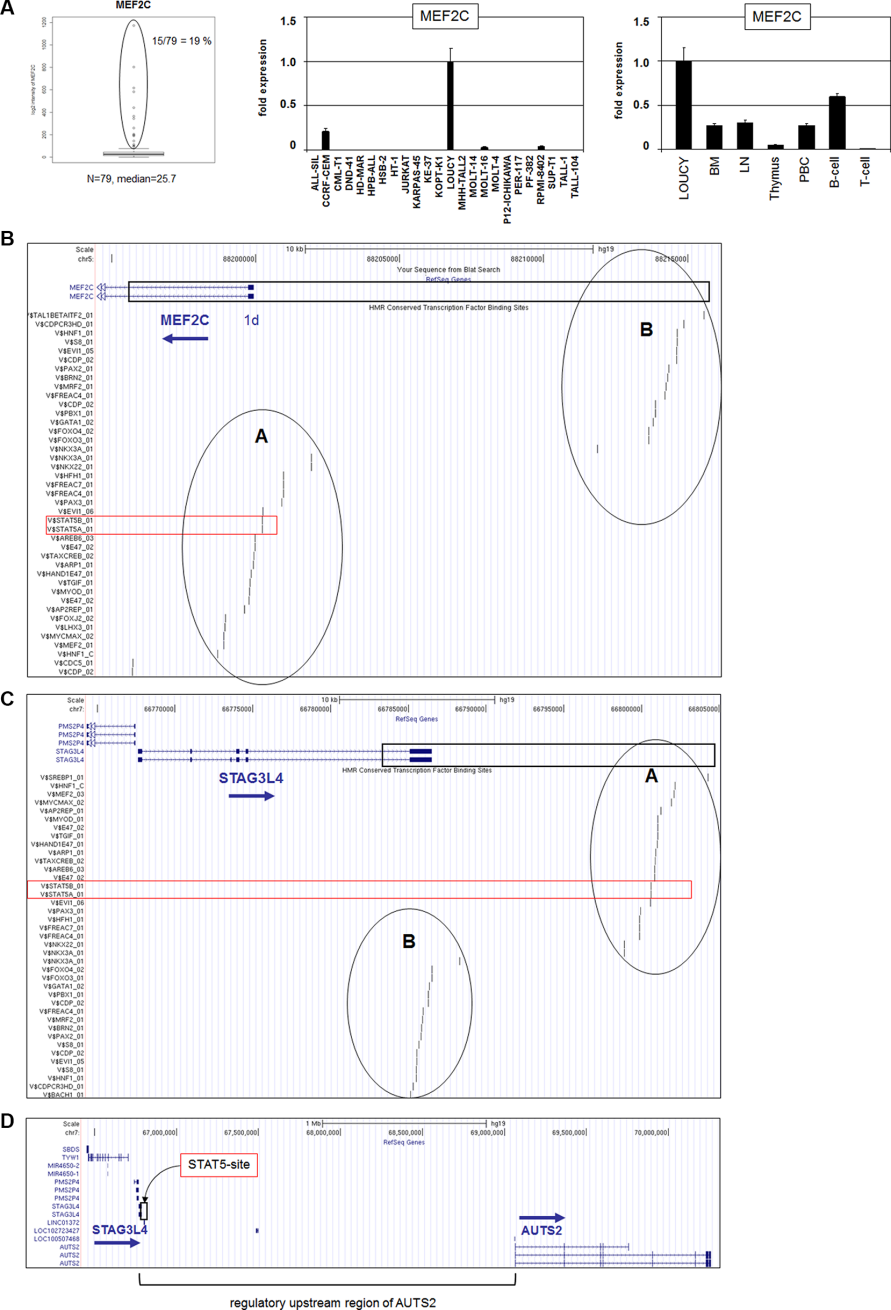
MEF2C expression and genomic regions at 5q14 and 7q11 (**A**) Expression analysis of MEF2C in T-ALL patients (public dataset GSE42038) indicated aberrant overexpression in 19% samples (left). RQ-PCR analysis of MEF2C in T-ALL cell lines demonstrated aberrant expression in 4 cell lines (middle). RQ-PCR analysis of MEF2C in primary cells showed high expression levels in B-cells, silencing in T-cells, and aberrant expression in T-ALL cells (right). (**B**–**D**) The 5q14 and 7q11 homologous sequence (boxed region) is located in the upstream region of MEF2C (B), in the downstream region of STAG3L4 (C) or in the upstream region of AUTS2 (D). This region contains two blocks of conserved TF binding sites which are named A and B (data were obtained from UCSC Genome Bioinformatics). The conserved STAT5 binding site is indicated in red.

Expression analysis of MEF2C in 79 pediatric T-ALL samples (public dataset GSE42038) demonstrated aberrant overexpression in 19% of the patients at varying levels (Figure 1A). RQ-PCR analysis of T-ALL cell lines yielded similar expression data for MEF2C in both frequency (4/24, 17%) and range of levels (Figure 1A). MOLT-16 and RPMI-8402 expressed low RNA levels while del(5q14) containing cell line LOUCY and NKX2-5 expressing cell line CCRF-CEM transcribed the highest levels, supporting the published data [26–28]. Quantification of MEF2C expression levels in primary hematopoietic cell/tissue samples in comparison to T-ALL cell line LOUCY showed vanishingly low expression in T-cells, contrastingly high levels in B-cells and aberrant activity in T-ALL (Figure 1A), endorsing these cell lines as suitable models for analyzing MEF2C deregulation in T-ALL. To investigate whether mutations in the reported repressive STAT5 TFBS located in the promoter region of MEF2C were implicated in T-cell leukemia, we performed sequence analyses of a 750 bp stretch in seven selected T-ALL cell lines (CCRF-CEM, JURKAT, MOLT-16, LOUCY, PEER, PER-117, RPMI-8402). However, this exercise showed the absence of mutations (data not shown), discounting involvement of sequence alterations in this TFBS in MEF2C deregulation in these T-ALL models.

BLAST and BLAT alignments of our obtained cell line data indicated corresponding sequences at 5q14 (MEF2C) and surprisingly at 7q11, showing few mismatched positions between these loci (Supplementary Figure S1). Extended analysis demonstrated that this sequence match between 5q14 and 7q11 covers a length of 19.15 kb (Figure 1B, 1C, 1D). Within these regions we recognized two blocks of conserved TFBS, named here A and B (Figure 1B, 1C). The indicated repressive binding site for STAT5 at 7q11 was located in block A, downstream of STAG3L4, respectively far upstream of AUTS2 (Figure 1D) which, therefore, might be involved in regulation of either of these genes.

Genomic comparisons between human and mouse showed structural differences at 7q11 but not at 5q14 (Supplementary Figure S2A, S2B). The absence of the conserved regulatory region together with STAG3L4 in the mouse genome likely reflects ancient processes of genomic evolution. Moreover, remaining STAG3-like genes are present in humans and primates but not in mice. These non-coding genes of unknown function probably emerged in higher vertebrates [31]. Furthermore, while STAG3L4 is located upstream of AUTS2 in humans it lies downstream of AUTS2 and WBSCR17 in the chimp (Supplementary Figure S2C), demonstrating ongoing alterations in this genomic region during primate evolution.

### Expression analysis of STAG3L4 and AUTS2

**Figure 2:**
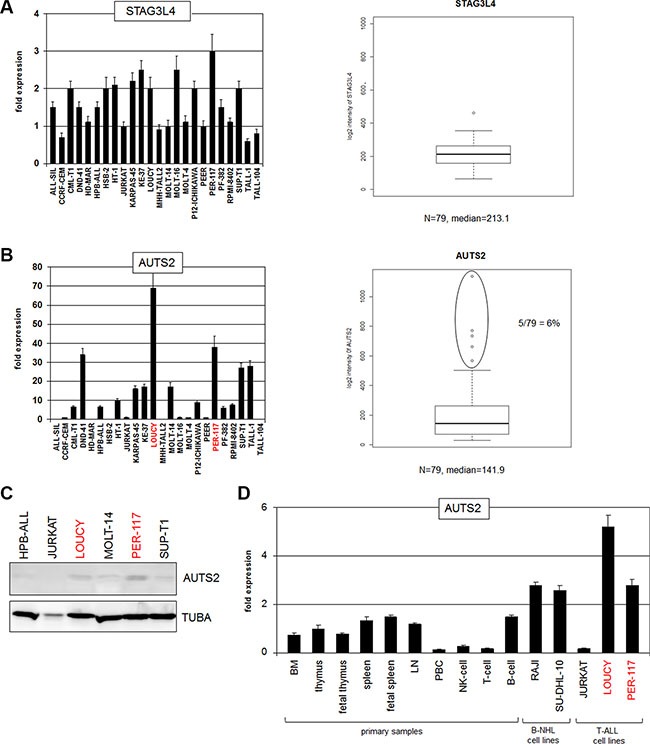
Expression analyses of STAG3L4 and AUTS2 (**A**) RQ-PCR analysis of STAG3L4 in T-ALL cell lines (left). Expression analysis of STAG3L4 in T-ALL patient samples (public dataset GSE42038) (right). (**B**) RQ-PCR analysis of AUTS2 in T-ALL cell lines indicated overexpression in 3 cell lines (left). Expression analysis of AUTS2 in T-ALL patients (GSE42038) indicated aberrant overexpression in 6% of the samples (right). (**C**) Western blot analysis of AUTS2 in selected T-ALL cell lines shows elevated expression in LOUCY and PER-117. Tubulin A served as loading control. (**D**) RQ-PCR analysis of AUTS2 in primary cells showed high expression levels in B-cells, reduced levels in T-cells and overexpression in LOUCY and PER-117.

Aberrant activation of MEF2C in T-ALL via chromosomal translocation or genomic deletion at 5q14 implicates the removal of its regulatory upstream region in the pathogenesis of this malignancy [26–28]. The observed similarity of regulatory sequences between chromosomal positions at 5q14 and 7q11 might indicate a role of these sequences in gene deregulation at 7q11 as well. Therefore, we asked if STAG3L4 and/or AUTS2 show aberrant expression patterns in T-cell leukemia. To this end we performed RQ-PCR and expression profiling analyses. Transcript levels of STAG3L4 showed similar values in all 24 T-ALL cell lines excluding aberrant expression in these samples (Figure 2A). Expression analysis of STAG3L4 in 79 T-ALL patients (GSE42038) again showed inconspicuous activity (Figure 2A), leaving this gene without pathological findings.

In contrast, we observed conspicuous discrepancies in AUTS2 transcript levels, demonstrating overexpression in T-ALL subsets of both cell lines and patients (Figure 2B, 2C). T-ALL cell lines LOUCY and PER-117 expressed the highest levels as shown by quantitative RQ-PCR and Western blot. Of note, these cell lines represent very early staged T-ALL cases which might reflect pathological significance [9, 18]. Among T-ALL patient samples analyzed, 6% showed elevated AUTS2 levels, pinpointing aberrant expression in a considerable subset of this malignancy. RQ-PCR analysis of AUTS2 in primary hematopoietic cells/tissues showed raised expression levels in B-cells and suppression in T-cells, indicating contrasting regulation in the development of lymphoid lineages (Figure 2D). Taken together, we identified physiological expression in hematopoiesis and aberrant activation of AUTS2 in a T-ALL subset. These findings prompted examinations of the regulation and function of AUTS2 in T-ALL in more detail, using high and low level expressing cell lines as models.

### (De)regulation of AUTS2 expression in T-ALL

**Figure 3:**
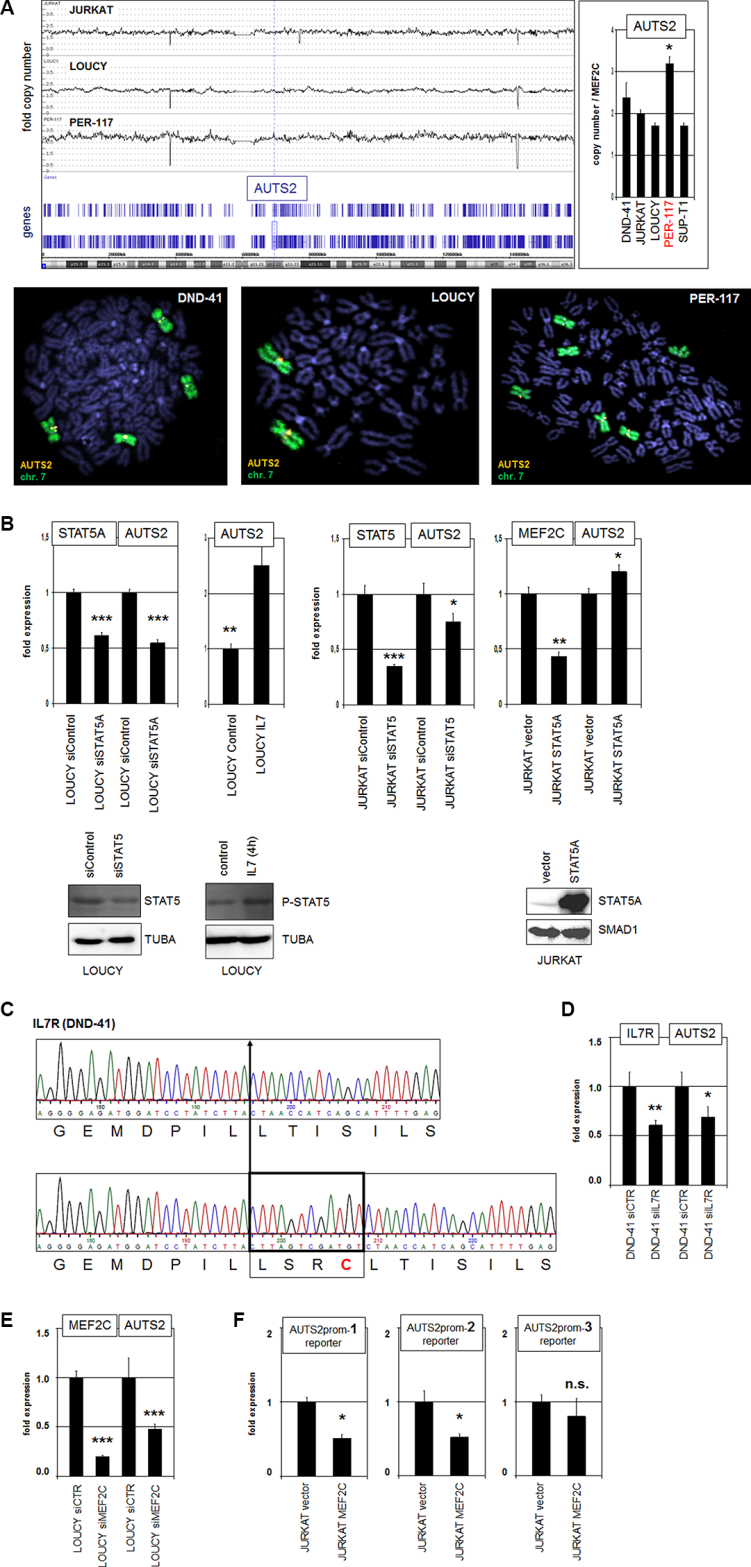
Gain at 7q11, IL7-STAT5 and MEF2C activate AUTS2 expression (**A**) Genomic profiling of T-ALL cell lines JURKAT, LOUCY and PER-117 shows copy number states of chromosome 7, indicating whole chromosome triploidy in PER-117 (above, left). Quantitative PCR analysis of AUTS2 indicated 3 copies in PER-117 (Above, right). FISH analysis of AUTS2 (orange) and chromosome 7 (green) in DND-41, LOUCY and PER-117 indicates absence of chromosomal rearrangements at AUTS2 (below). (**B**) RQ-PCR analysis (above) after siRNA-mediated knockdown of STAT5, treatment with IL7, and forced overexpression of STAT5 in LOUCY and JURKAT cells. Western blot analyses (below) confirmed knockdown, increased phosphorylation and overexpression of STAT5. Tubulin A and SMAD1 served as loading controls. (**C**) Sequencing results of a IL7R gene section in DND-41 cells show wild type configuration of one allele (above) and an insertion in the other allele (below). This insertion encodes 4 amino acid residues comprising cysteine which mediates aberrant receptor activation. (**D**) SiRNA-mediated knockdown of IL7R in DND-41 resulted in reduced expression of AUTS2. (**E**) SiRNA-mediated knockdown of MEF2C in LOUCY resulted in reduced expression of AUTS2. (**F**) Reporter gene assay analyzing 3 potential MEF2C binding sites in the upstream region of AUTS2 demonstrated direct binding and regulation of AUTS2 by MEF2C.

The genomic region at 7q11 in humans showed several alterations in comparison to other vertebrates (see above). Moreover, chromosomal rearrangements of AUTS2 have been described in neurodevelopmental disorders and B-cell precursor ALL [32–36]. To investigate the role of genomic alterations of AUTS2 in T-ALL we performed genomic array analyses of T-ALL cell lines CCRF-CEM, JURKAT, LOUCY, PER-117 and RPMI-8402. These data indicated a copy number gain at 7q11 only in PER-117 which exhibits chromosome 7 triploidy (Figure 3A). RQ-PCR analysis of genomic DNA from T-ALL cell lines DND-41, JURKAT, LOUCY, PER-117 and SUP-T1 demonstrated alone for PER-117 three copies of AUTS2, confirming the array data and excluding cryptic amplifications (Figure 3A). FISH analyses of the AUTS2 locus in DND-41, LOUCY and PER-117 showed absence of chromosomal translocations (Figure 3A), indicating that genomic aberrations do not play a major role in AUTS2 activation in T-ALL.

Next, we analyzed candidate TFs which might mediate AUTS2 (de)regulation in T-ALL cells. As described above, we identified a conserved STAT5-site located in the far upstream region of AUTS2 (Figure 1C). To check if STAT5 regulates AUTS2 transcription we performed in LOUCY and JURKAT cells siRNA-mediated knockdown and forced overexpression of STAT5, as well as treatment with STAT5-activator IL7 (Figure 3B). Collectively, these data showed that STAT5 activates transcription of AUTS2. Recently, mutations in the extracellular juxta-transmembrane domain of the IL7 receptor gene IL7R have been shown to drive constitutive signalling activity via STAT5 in T-ALL [37]. Sequence analysis of this gene section in IL7R-expressing T-ALL cell lines detected the reported insertion-type of mutation in one IL7R allele of DND-41 (Figure 3C). Accordingly, siRNA-mediated knockdown of IL7R in DND-41 resulted in reduced expression of AUTS2 (Figure 3D), explaining elevated AUTS2 levels in this cell line (Figure 2B), and supporting that IL7-STAT5-signalling mediates AUTS2 activation.

Furthermore, in silico sequence analysis of the AUTS2 promoter region revealed three MEF2C binding sites located at −2808 bp, −5527 bp, and −24537 bp (Supplementary Figure S3). To see if MEF2C regulates AUTS2 expression we performed siRNA-mediated knockdown of MEF2C and quantified AUTS2 transcript levels. These data demonstrated that MEF2C mediated AUTS2 activation in LOUCY cells (Figure 3E). Additionally, we performed reporter gene assays for each of these MEF2C sites in JURKAT cells. While forced expression of MEF2C together with a reporter-construct containing distal binding site 3 showed no significant effect, constructs containing sites 1 or 2 were accompanied by reporter gene inhibition, showing that MEF2C directly binds and regulates AUTS2 via these two proximal sites (Figure 3F). However, the observed reporter gene inhibition instead of an expected activation may result from cloning constrictions of the regulatory regions artificially performed for this assay. Taken together, we showed that copy number gain, IL7-STAT5-signalling, and TF MEF2C contribute to the aberrant expression of AUTS2 in T-ALL.

### AUTS2 activates expression of NKL homeobox gene MSX1

**Figure 4:**
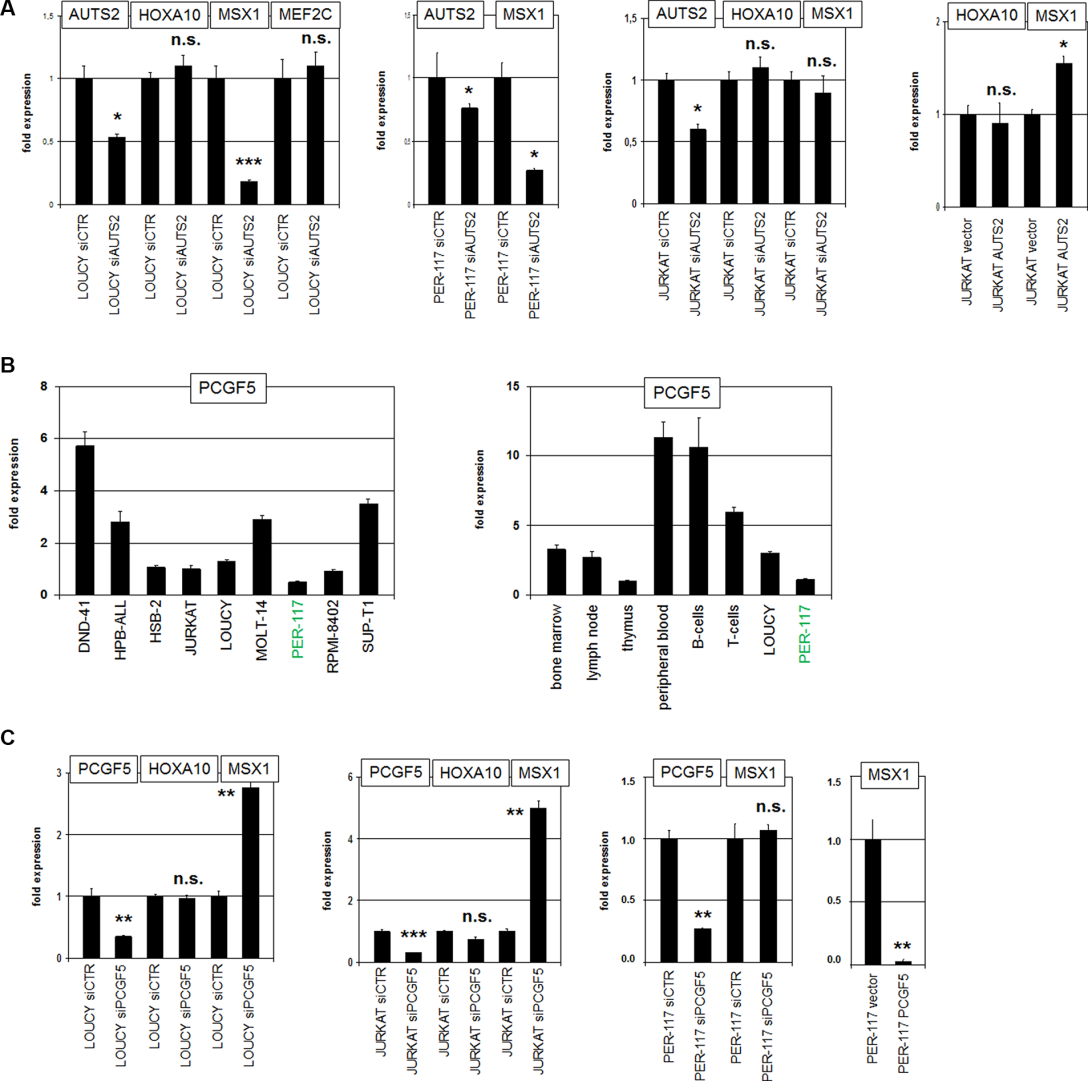
AUTS2 and PCGF5 regulate MSX1 expression (**A**) SiRNA-mediated knockdown of AUTS2 in LOUCY resulted in reduced expression of MSX1 but not of HOXA10 or MEF2C (left). SiRNA-mediated knockdown of AUTS2 in PER-117 resulted in reduced expression of MSX1 (middle). SiRNA-mediated knockdown of AUTS2 in JURKAT showed no change in MSX1 expression levels (middle). Forced expression of AUTS2 in JURKAT resulted in MSX1 upregulation while HOXA10 levels remained unaffected (right). (**B**) RQ-PCR analysis of PCGF5 in selected T-ALL cell lines (left). PER-117 showed the lowest PCGF5 expression level. RQ-PCR analysis of PCGF5 in primary hematopoietic cells showed elevated expression levels in B- and T-cells and reduced levels in T-ALL cell lines (right). (**C**) SiRNA-mediated knockdown of PCGF5 in LOUCY (left) and JURKAT (middle) resulted in enhanced expression of MSX1 but not of HOXA10. SiRNA-mediated knockdown of PCGF5 in PER-117 (middle) left MSX1 expression unperturbed. Forced expression of PCGF5 in PER-117 resulted in reduced expression levels of MSX1 (right).

AUTS2 encodes a nuclear protein which interacts with PCGF5/PRC1.5 and converts this subtype of repressor complex into an activator [38, 39], indicating that the AUTS2 regulated genes are enhanced in overexpressing cells. To identify AUTS2 target genes in T-ALL we performed expression profiling. Comparing AUTS2-high (LOUCY, PER-117) and AUTS2-low (ALL-SIL, CCRF-CEM, PEER) T-ALL cell lines we found 2166 gene candidates showing at least 6-fold enhanced expression levels (Supplementary Table S1). This approach confirmed elevated expression of AUTS2 and MEF2C and revealed upregulation of several homeobox genes including members of the HOXA- and HOXB-cluster, MSX1 and PBX1. Accordingly, gene-annotation enrichment analysis of the top 500 differentially expressed genes indicated that AUTS2 regulates developmental processes including transcription and signal transduction (data not shown). We focused on MSX1 and HOXA10 previously described as aberrantly expressed in early-staged T-ALL and which are regulated by chromatin modifications [9, 13, 18]. SiRNA-mediated knockdown of AUTS2 in LOUCY resulted in reduced expression of MSX1 while HOXA10 and MEF2C levels did not change (Figure 4A). AUTS2-knockdown in PER-117 resulted in MSX1 reduction as well, while in AUTS2-low cell line JURKAT no change was effected by this treatment (Figure 4A). In contrast, forced expression of AUTS2 in JURKAT cells resulted in elevated MSX1 expression (Figure 4A), supporting an activatory input of AUTS2 on homeobox gene MSX1.

Quantification of PCGF5 transcripts in T-ALL cell lines by RQ-PCR indicated the highest values for DND-41 and the lowest for PER-117 (Figure 4B), showing an inverse relation of expression levels for repressor gene PCGF5 and homeobox gene MSX1 [9]. Analysis of PCGF5 transcription in primary hematopoietic cells/tissues demonstrated enhanced values in both B-cells and T-cells, indicating upregulation of PCGF5 expression during lymphoid development (Figure 4B). Comparison of PCGF5 transcript levels between LOUCY, PER-117 and primary hematopoietic cells showed reduced expression in both T-ALL cell lines (Figure 4B). SiRNA-mediated knockdown of PCGF5 resulted in increased expression of MSX1 in LOUCY and JURKAT but not in PER-117 (Figure 4C). In contrast, forced expression of PCGF5 in PER-117 resulted in strongly decreased MSX1 expression (Figure 4C), supporting that PCGF5 repressed MSX1 and suggesting that the relative amounts of AUTS2 and PCGF5 may play a key role in target gene regulation. Again HOXA10 expression was not affected by PCGF5 knockdown (Figure 4C), discounting PCGF5/PRC1.5 as a general regulator of homeobox genes.

**Figure 5:**
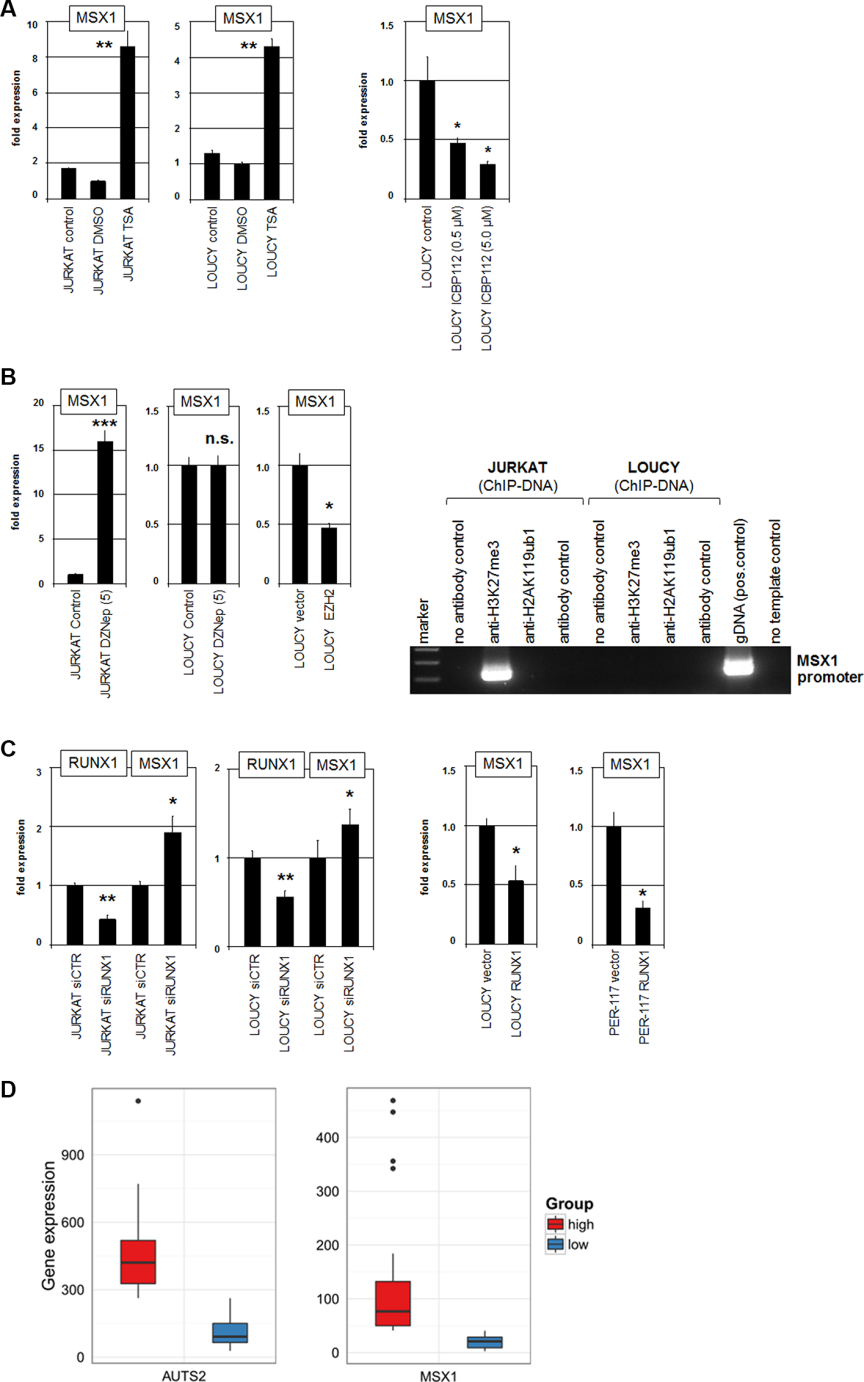
PRC1, PRC2 and RUNX1 regulate MSX1 (**A**) RQ-PCR analysis of MSX1 in JURKAT and LOUCY after treatment with HDAC-inhibitor TSA resulted in enhanced expression levels (left). RQ-PCR analysis of MSX1 in LOUCY after treatment with EP300-inhibitor ICBP112 resulted in reduced expression levels (middle). (**B**) RQ-PCR analysis of MSX1 in JURKAT and LOUCY after treatment with EZH2-inhibitor DZNep resulted in enhanced expression levels in JURKAT but showed no change in EZH2-negative LOUCY (left). Forced expression of EZH2 in LOUCY resulted in reduced expression of MSX1 (left). ChIP analysis of H3K27me3 and H2A119ub1 in JURKAT and LOUCY demonstrated H3K27-trimethylation at MSX1 in JURKAT but not in LOUCY (right). (**C**) SiRNA-mediated knockdown of RUNX1 in JURKAT and LOUCY resulted in increased expression of MSX1 (left). In LOUCY and PER-117 forced expression of RUNX1 mediated decreased MSX1 expression (right). (**D**) Expression analysis of AUTS2 (left) and MSX1 (right) in T-ALL patients (public dataset GSE42038) demonstrated that the overlap of patients with maximal RNA expression of both AUTS2 and MSX1 was statistically significant (*p* = 0.0053, hypergeometric test, *n* = 20 draws of *N* = 79 patients), supporting the observed activating impact of AUTS2 on MSX1 expression.

PRCs mediate gene repression via modification of histones by methylation and ubiquitinylation [15]. AUTS2 inhibits histone ubiquitinylation and recruits histone acetyltransferase EP300 to effect gene activation [39]. Accordingly, treatment with histone deacetylase inhibitor TSA resulted in increased MSX1 expression and treatment with EP300 inhibitor ICBP112 resulted in decreased expression (Figure 5A). These data demonstrate that histone acetylation is involved in the transcriptional regulation of NKL homeobox gene MSX1.

Furthermore, PRC1 interacts with PRC2 leading to combined or sequential operations in gene suppression [40, 41]. EZH2 represents the central component of PRC2 and performs repressive tri-methylation of histone H3 at K27 [15]. DZNep is a pharmacological inhibitor which mediates degradation of EZH2 as shown recently in JURKAT cells [18, 42]. Here, treatment of JURKAT cells with DZNep resulted in enhanced expression of MSX1 (Figure 5B), indicating a suppressive impact of PRC2 on this homeobox gene as well. LOUCY does not express EZH2 [18], consistently showing no effect of DZNep treatment on MSX1 expression (Figure 5B). In contrast, forced expression of EZH2 in LOUCY cells resulted in reduced transcription of MSX1 (Figure 5B), supporting that EZH2/PRC2 mediates repression of MSX1. Supporting this notion, ChIP analysis demonstrated the presence of H3K27me3 at the promoter region of MSX1 in JURKAT but not in LOUCY cells (Figure 5B). However, we were unable to detect H2AK119ub1 at that region, leaving open whether PRC1-mediated histone H2A ubiquitinylation plays a role in MSX1 regulation in these cells.

Gene-specific recruitment of PRC1 is performed inter alias by binding to particular TFs. The hematopoietic TF RUNX1 has been shown to interact/recruit PRC1 components including PCGF5 [43]. Sequence-analysis of the MSX1 promoter region revealed two potential binding sites for RUNX1 located at −474 bp and −1576 bp (Supplementary Figure S4), suggesting that RUNX1 may aid the recruitment of repressor complex PRC1.5 to the regulatory region of MSX1. To investigate the impact of RUNX1 on the expression of MSX1 we performed siRNA-mediated knockdown in JURKAT and LOUCY cells. This treatment resulted in elevated expression of MSX1 (Figure 5C). Moreover, forced expression of RUNX1 in LOUCY and PER-117 resulted in reduced MSX1 transcription (Figure 5C), consistent with transcriptional inhibition by RUNX1.

Finally, gene analyses of 79 T-ALL patients (GSE42038) demonstrated that the overlap of patients with maximal RNA expression of both AUTS2 and MSX1 was statistically significant (*p* = 0.0053) (Figure 5D). These data support that our experimental findings obtained in T-ALL cell lines have clinical significance. Therefore, T-ALL patients showing upregulation of AUTS2 or MSX1 may benefit from treatments with specific inhibitors of chromatin regulators, representing a promising therapeutic approach for this subset of patients.

## DISCUSSION

**Figure 6:**
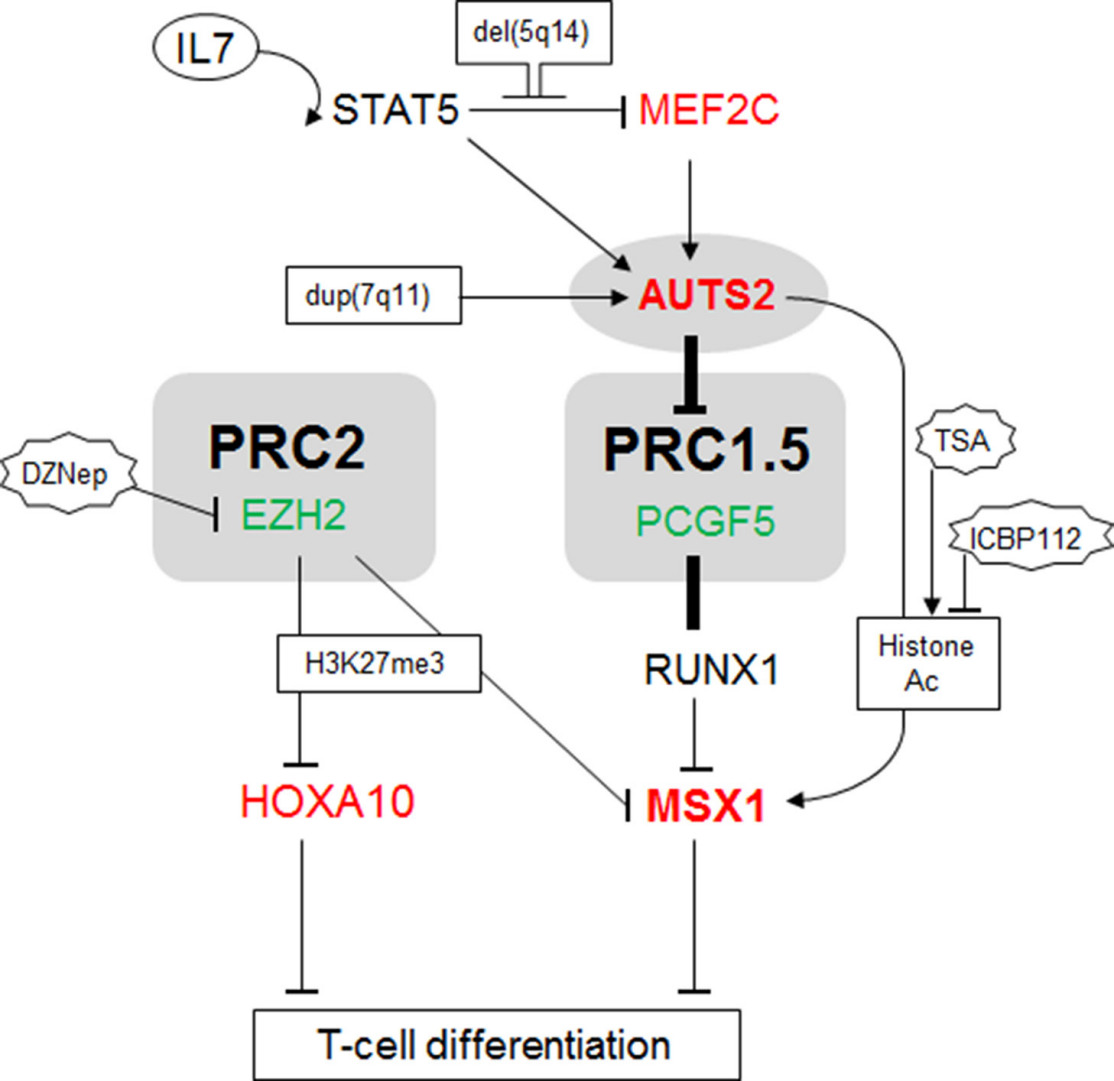
Gene regulatory network comprising AUTS2 and MSX1 This figure summarizes the results obtained in this study. IL7-STAT5-signalling is located upstream of MEF2C and AUTS2. AUTS2 interacts with PCGF5/PRC1.5 turning the repressive impact of this complex into an activatory, resulting in elevated expression of MSX1. PRC2 mediates repressive H3K27-trimethylation and AUTS2 activatory histone-acetylation. MSX1 is regulated by PRC1.5 and PRC2 while HOXA10 is regulated just by PRC2. Both, HOXA10 and MSX1 are involved in lymphoid differentiation.

Our key findings are summarized in Figure 6, specifically the identification of AUTS2 and PCGF5 as antagonistic regulators of NKL homeobox gene MSX1 in T-ALL cells. We also showed that MSX1 is repressed by EZH2 via tri-methylation of histone H3 and that histone acetylation activates MSX1 transcription probably via histone acetyltransferase EP300 recruitment by AUTS2. Instead of chromosomal rearrangement AUTS2 deregulation is conducted by the IL7-STAT5-pathway and by MEF2C. STAT5 activates AUTS2 but simultaneously represses MEF2C. The corresponding STAT5 binding sites are embedded in large regulatory upstream regions which are conserved between AUTS2 at 7q11 and MEF2C at 5q14. Our data suggest synergistic inputs of AUTS2 and MEF2C in lymphopoiesis and leukemia (de)regulating NKL homeobox gene MSX1.

Genomic comparisons between human and mouse revealed similar gene configurations at 5q14 but several differences at 7q11. Humans possess 6 gene variants of STAG3 (STAG3L1-6) absent in the mouse genome. STAG3 encodes a meiotic protein while the function of the non-coding STAG3-like RNAs is elusive [31, 44]. AUTS2 encodes an evolutionary conserved nuclear protein [38]. Mutations of AUTS2 have been correlated with neurodevelopmental disorders [33]. Accordingly, AUTS2 is expressed in fetal brains but also in leukocytes [32, 33]. Interestingly, the human AUTS2 gene differs from its orthologue in Neandertals, indicating ongoing evolutionary alterations in this chromosomal region [45]. Genomic aberrations targeting AUTS2 have been reported in patients with neurodevelopmental disorders and B-cell precursor ALL while absent from T-ALL as shown here [32–36]. Aberrant fusions with the B-cell specific gene PAX5 may indicate physiological expression and function of AUTS2 in B-cell development. Accordingly, our data demonstrate AUTS2 activity in B-cells and silenced transcription in T-cells, indicating lineage-specific functions in lymphopoiesis. Aberrant reactivation in the T-cell lineage may thus promote developmental defects or leukemic transformation.

STAT5 and MEF2C regulate AUTS2 transcription in T-ALL (as shown here) and are coexpressed in the fetal mouse brain (Supplementary Figure S5). MEF2C is a key developmental factor in brain neurogenesis and deletions have been reported in autism-related disorders [46, 47]. Furthermore, left/right asymmetry in the developing brain reportedly correlates with AUTS2 and MEF2C expression [48]. MEF2C is also expressed in B-cell but not in T-cell development [23, 25], thus correlating with AUTS2 activity in B-cell lymphopoiesis. In T-ALL both MEF2C and STAT5 are deregulated and contribute to leukemogenesis. MEF2C serves as an oncogene in an immature subtype of T-ALL and is aberrantly activated via NKL homeodomain TF NKX2-5 or by genomic upstream-deletions eliminating a repressive binding site for STAT5 [26–28]. STAT5 is mobilized via the IL7-pathway which is constitutively activated by particular mutations in the IL7-receptor [37]. Here, we identified an insertion-type mutation in one allele of the IL7R gene of the T-ALL cell line DND-41 which mediated transcriptional activation of AUTS2. This cell line additionally expresses NKL homeobox gene TLX3, supporting the described correlation between IL7R and TLX3 in T-ALL patients [37]. Our data demonstrated AUTS2 as target gene of this aberrantly activated pathway. Thus, a network linking MEF2C, IL7R, STAT5 and AUTS2 may play a role in two cellular contexts: T-cell differentiation/leukemia and brain development/autism-related disorders.

AUTS2 protein interacts with a particular type of PRC1 multiprotein complex via the component PCGF5. This interaction turns the repressor PRC1.5 into a gene activating complex via inhibition of histone ubiquitinylation and recruitment of histone acetyltransferase EP300 [39]. The hematopoietic TF RUNX1 has been shown to interact with PCGF5 as well and may guide the positioning of PCR1.5 to specific target genes including MSX1 [43]. Our data indicate that the balance of PCGF5 and AUTS2 is important for regulation of target gene activity. We observed overexpression of AUTS2 and decreased levels of PCGF5 in T-ALL subsets. In contrast, overexpression of PCGF5 has been reported for esophageal adenocarcinoma, indicating that PCR1.5 may activate or suppress tumorigenesis, depending on the cellular context [49].

Polycomb repressor complexes regulate many developmental genes including homeobox genes. Six subtypes of PRC1 have been described which differ in their PCGF-component [16]. PCGF1 containing complex PRC1.1 suppresses HOXA genes including HOXA10 in hematopoiesis [50]. Our data showed that PCGF5/PRC1.5 repressed MSX1 but not HOXA10, demonstrating target gene specific differences between these complexes. We also showed that MSX1 was suppressed by PRC2 while activated by histone acetylation. PRC1 and PRC2 cooperate in gene regulation [40, 41]. However, the exact mode of their impact on MSX1 remains unclear. We detected PRC2-mediated H3K27me3 but no H2A119u1 at the target gene MSX1, suggesting that PRC1.5 performs MSX1 suppression without histone-ubiquitinylation as described for other PRC1-regulated homeobox genes as well [14, 40, 51].

The expression of MSX1 is controlled by methylation and acetylation of histones in other tissues as well, indicating that the modulation of its chromatin structure represents a fundamental mode of transcriptional regulation. In the embryonal neural crest border region histone methyltransferase NSD3 activates MSX1 transcription probably via H3K4-methylation [52, 53]. In myoblasts and neural crest cells HDAC1 and HDAC3, respectively, inhibits MSX1 expression demonstrating an activating input of histone acetylation in these cell types [54, 55]. On the other hand, MSX1 mediates target gene regulation via cooperation with particular chromatin-proteins. MSX1 protein interacts with histone H1, PRC2 components SUZ12 and EED, and with histone methyltransferase G9A, demonstrating mutual regulation of and by MSX1 via repressive chromatin components [13, 56, 57].

MSX1 represents a primarily physiological member of the NKL subclass of homeobox genes in lymphopoiesis [9, 10, 58]. While several oncogenic NKL homeobox genes are activated by chromosomal rearrangements in T-ALL, MSX1 has been shown to be overexpressed in T-ALL subsets via aberrantly reduced BMP-signalling [9, 59]. Here, we have shown that activation of MSX1 via AUTS2 overexpression represents an additional mechanism of its deregulation in T-ALL. Moreover, our data indicate that AUTS2 may represent a physiological regulator in B-cell differentiation. The situation that mutated/deregulated PRC-components play a role in ETP-ALL as well as in immature T-ALL as described here supports their reported overlapping gene signature and subsequent membership in a single disease entity [60]. Early-staged T-cell malignancies are connected with bad outcome and require additional efforts to develop novel therapeutic strategies [20, 21, 61]. The immature T-ALL cell lines LOUCY and PER-117 described in this study may represent suitable preclinical models for this task.

## MATERIALS AND METHODS

### Sequence analysis

Genomic DNA from T-ALL cell lines was isolated using the DNeasy Blood and Tissue Kit (Qiagen, Hilden, Germany). Amplification of MEF2C upstream regions containing the described STAT5 binding site in addition to flanking regions was performed by polymerase chain-reaction (PCR) [28], using the thermocycler TGradient (Biometra, Göttingen, Germany) and the following oligonucleotides: MEF2C-for 5′-AAGAGAACTCAAGCTTTAGCCAG-3′, and MEF2C-rev 5′-GGGCTGCGTTTGCCTCCTCTCC-3′ (Eurofins MWG, Ebersbach, Germany). The PCR product (750 bp) was cloned into pGEM-T Easy (Promega, Mannheim, Germany) and sequenced at Eurofins MWG. The obtained sequence data were analyzed by BLAST (blast.ncbi.nlm.nih.gov) and BLAT (genome.cse.ucsc.edu).

Cloning of the analyzed IL7R gene region was performed using oligonucleotides IL7R-for 5′-TGCTCCAACCGGCAGCA-3′ and IL7R-rev 5′-CCCTATGAATCTGGCAGTCC-3′ (Eurofins MWG). The PCR product (351 bp) was cloned into pGEM-T Easy (Promega) and sequenced at Eurofins MWG.

### Real-time quantitative polymerase chain-reaction (RQ-PCR) analyses

Total RNA was extracted from cell line samples using TRIzol reagent (Invitrogen, Darmstadt, Germany). Primary human total RNA used in this study was commercially obtained - isolated from peripheral blood mononuclear cells (PBC), thymus, fetal thymus, lymph node (LN), spleen, fetal spleen, and bone marrow (BM) from Biochain/BioCat (Heidelberg, Germany), and RNA from peripheral CD19-positive B-cells and CD3-positive T-cells from Miltenyi Biotec (Bergisch Gladbach, Germany). cDNA was synthesized from 5 μg RNA by random priming using Superscript II (Invitrogen/Thermo Fisher, Darmstadt, Germany). RQ-PCR analysis was performed with the 7500 Real-time System, using commercial buffer and primer sets (Applied Biosystems/Life Technologies, Darmstadt, Germany). Quantification of MSX1 was performed as described recently [9]. For normalization of expression levels we analyzed the transcript of TATA box binding protein (TBP).

For genomic copy number quantification we designed oligonucleotides hybridizing within the coding region of the AUTS2 gene: AUTS2-for 5′-GAACCATGGATGGCCCGACG-3′, AUTS2-rev 5′-GACGACGAGGCGAGTGAGAG-3′. As reference we used the MEF2C locus as reported previously [NAGEL 2011].

Quantitative analyses were performed in triplicate. Standard deviations are presented in the figures as error bars. The statistical significance was assessed by Student's T-Test and the calculated *p*-values are indicated by asterisks (*p < 0.05, **p < 0.01, ***p < 0.001, n.s. not significant).

### Expression profiling

Gene expression microarray profiling data of cell lines were generated using the HG U133 Plus 2.0 gene chip (Affymetrix, High Wycombe, UK). Datasets for T-ALL cell lines ALL-SIL, CCRF-CEM, LOUCY and PEER were generated by Prof. Andreas Rosenwald (Institute of Pathology, University of Würzburg, Germany), datasets for T-ALL cell line PER-117 by Dr. Robert Geffers (Genome Analytics Facility, Helmholtz Centre for Infection Research, Braunschweig, Germany). After RMA-background correction and quantile normalization of the spot intensities, the data were expressed as ratios of the sample mean and subsequently log2 transformed. Data processing was done via R/Bioconductor using limma and affy packages (http://www.bioconductor.org/), and data analysis performed using Microsoft Excel. For comparative expression profiling analysis the median values of AUTS2-high and AUTS2-low cell lines were subtracted and ordered. A cut-off at 2.6 represents a minimum of 6-fold expression difference (Supplementary Table S1). To parse biological function of shortlisted genes, gene-annotation enrichment analysis was performed using DAVID bioinformatics resources [62].

The public GEO dataset GSE42038 was used for expression analyses of 79 T-ALL patients [Horstmann and Otto, unpublished]. The samples represent naïve primary T-ALLs from children. The dataset was generated using the HG U133 Plus 2.0 gene chip platform from Affymetrix and deposited at the National Center for Biotechnology Information (www.ncbi.nlm.nih.gov). Supplemented online tools were used for data analysis and R-based protocols for statistical calculations and data illustrations by boxplots (http://www.cran.r-project.org/). Gene overexpression in the patient dataset was determined as percent of outliers visualized in boxplots. Outliers are located outside of the 1.5 interquartile range (IQR) of the upper quartile.

### Chromosomal and genomic analyses

Chromosomal analysis by fluorescent *in situ* hybridization (FISH) was performed as described previously [63]. RP11-BAC clones were obtained from BacPac Resources, Children's Hospital Oakland Research Institute (CA, USA), insert DNA harvested using the Big BAC DNA Kit (Princeton Separations, Adelphia, NJ, USA) and directly labelled by nick translation with dUTP-fluors (Dyomics, Jena, Germany). Whole chromosome paint probes were obtained from Applied Spectral Imaging (Neckarhausen, Germany). Fluorescent images were captured and analyzed with an Axio-Imager microscope (Zeiss, Göttingen, Germany) configured to a dual Spectral Imaging FISH system (Applied Spectral Imaging, Neckarhausen, Germany).

For genomic profiling cell line genomic DNA was prepared by the Qiagen Gentra Puregene Kit (Qiagen). Genomic profiling was performed at the Genome Analytics Facility, Helmholtz Centre for Infection Research (Braunschweig, Germany). Labeling, hybridization and washing was performed using CytoScan HD arrays and the recommended kits according to the manufacturers protocols (Affymetrix). Data were subsequently transformed and analyzed using the Chromosome Analysis Suite software version 3.1.0.15 (Affymetrix).

### Cell lines and treatments

T-ALL cell lines are held by the DSMZ (Braunschweig, Germany) and were cultivated as described previously [64]. PER-117 cells were kindly provided by Dr. Ursula Kees (Perth, Australia) [65]. Treatments of cell lines were performed for 20 h with 5 μM 3-Deazaneplanocin A hydrochloride (DZNep) (Sigma, Taufkirchen, Germany), 10 μg/ml Trichostatin A (TSA) (Sigma), indicated concentrations of ICBP112 (Sigma), or 20 ng/ml recombinant human Interleukin-7 (IL7) protein (R&D Systems, Minneapolis, MN, USA). Gene specific siRNA oligonucleotides and AllStars negative Control siRNA (siControl) were obtained from Qiagen. Expression constructs for AUTS2, PCGF5 and RUNX1 were obtained from Origene (Wiesbaden, Germany). SiRNAs (80 pmol) and expression constructs/vector controls (2 μg) were transfected into 1×10^6^ cells by electroporation using the EPI-2500 impulse generator (Fischer, Heidelberg, Germany) at 350 V for 10 ms. Electroporated cells were harvested after 20 h of cultivation. Lentiviral transduction of the STAT5 gene into JURKAT cells was performed as described previously [28, 66].

### Chromatin Immuno-Precipitation (ChIP)

ChIP analysis was performed with the ChIP Assay Kit (Millipore-Upstate, Schwalbach, Germany) according to the manufacturer protocol. Genomic fragments were generated by sonication. We used ChIP-validated antibodies anti-H3K27me3 and anti-ubiquityl-H2A (Millipore-Upstate). The subsequent nested PCR analysis of the isolated DNA fragments was performed using taqpol (Qiagen), the thermocycler TGradient (Biometra), and the following oligonucleotides (Eurofins MWG): MSX1-for 5′-CTCAGCATATCTCGGCGGCC-3′, MSX1-rev 5′-CTCACTCGGCCCTGCCATTG-3′, MSX1-for-nested 5′-TGGACAGATGGGAGGCTACC-3′, MSX1-rev-nested 5′-GGCTGCCGTGGCCATTTAGG-3′.

### Protein analyses

Western blots were generated by the semi-dry method. Proteins obtained from cell line lysates using SIGMAFast protease inhibitor cocktail (Sigma) were transferred onto nitrocellulose membranes (Bio-Rad, München, Germany) and blocked with 5% dry milk powder dissolved in phosphate-buffered-saline buffer (PBS). The following antibodies were used: alpha-Tubulin (Sigma), AUTS2 (Origene), SMAD1 (Santa Cruz Biotechnology, Heidelberg, Germany), STAT5 (Santa Cruz Biotechnology), phospho-STAT5 (Cell Signaling Technology, Danvers, MA, USA). For loading control the blots were reversibly stained with Poinceau (Sigma) and then detection of alpha-Tubulin (TUBA) or SMAD1 was performed. Secondary antibodies were linked to peroxidase for detection by Western-Lightning-ECL (Perkin Elmer, Waltham, MA, USA). Documentation was performed using the digital system ChemoStar Imager (INTAS, Göttingen, Germany).

### Reporter gene assay

For creation of reporter gene constructs we combined a reporter with three different regulatory genomic fragments derived from the upstream region of AUTS2, containing MEF2C binding sites. We cloned genomic PCR products of the corresponding upstream regions (regulators) and of the HOXA9 gene, comprising exon1-intron1-exon2 (reporter), into the *Hind*III/*Bam*HI and *Eco*RI sites, respectively, of the expression vector pcDNA3 downstream of the CMV enhancer [28]. The oligonucleotides used for the amplification of the AUTS2-regulators were obtained from Eurofins MWG. Their sequences were as follows: AUTS2-for1 5′-CCAAGCTTGTTGAAAGGTGACTGTAATTGC-3′, AUTS2-rev1 5′-AGGGATCCTGTGTTAACTTCTAGA GTGCTG-3′, AUTS2-for2 5′-CTAAGCTTTTTCCAAGT GAGCAAATAAGGAG-3′, AUTS2-rev2 5′-GGGGATCC TTAGGGCAAAGTTACAACAATG-3′, AUTS2-for3 5′-T GAAGCTTGTCAATAGTTCCTCTTGTGGTC-3′, AUT S2-rev3 5′-TTGGATCCTGGCACTTACTCTGTCCATT TC-3′. Introduced restriction sites used for cloning are underlined. Constructs were validated by sequence analysis (Eurofins MWG). Commercial HOXA9 and TBP assays were used for RQ-PCR to quantify the spliced reporter-transcript, corresponding to the regulator activity. A cotransfected commercial luciferase construct served as transfection control and was quantified by the Luciferase Assay System (Promega) using the luminometer Lumat LB9501 (Berthold Technologies, Bad Wildbad, Germany).

## SUPPLEMENTARY MATERIALS FIGURES AND TABLE





## References

[1] Ferrando AA, Look AT (2003). Gene expression profiling in T-cell acute lymphoblastic leukemia. Semin Hematol.

[2] Armstrong SA, Look AT (2005). Molecular genetics of acute lymphoblastic leukemia. J Clin Oncol.

[3] Peirs S, Van der Meulen J, Van de Walle I, Taghon T, Speleman F, Poppe B, Van Vlierberghe P (2015). Epigenetics in T-cell acute lymphoblastic leukemia. Immunol Rev.

[4] Yui MA, Rothenberg EV (2014). Developmental gene networks: a triathlon on the course to T cell identity. Nat Rev Immunol.

[5] Hatano M, Roberts CW, Minden M, Crist WM, Korsmeyer SJ (1991). Deregulation of a homeobox gene, HOX11, by the t(10; 14) in T cell leukemia. Science.

[6] Bernard OA, Busson-LeConiat M, Ballerini P, Mauchauffé M, Della Valle V, Monni R, Nguyen Khac F, Mercher T, Penard-Lacronique V, Pasturaud P, Gressin L, Heilig R, Daniel MT (2001). A new recurrent and specific cryptic translocation, t(5;14)(q35;q32), is associated with expression of the Hox11L2 gene in T acute lymphoblastic leukemia. Leukemia.

[7] MacLeod RA, Nagel S, Kaufmann M, Janssen JW, Drexler HG (2003). Activation of HOX11L2 by juxtaposition with 3′-BCL11B in an acute lymphoblastic leukemia cell line (HPB-ALL) with t(5;14)(q35;q32. 2). Genes Chromosomes Cancer.

[8] Nagel S, Kaufmann M, Drexler HG, MacLeod RA (2003). The cardiac homeobox gene NKX2-5 is deregulated by juxtaposition with BCL11B in pediatric T-ALL cell lines via a novel t(5;14)(q35.1;q32.2). Cancer Res.

[9] Nagel S, Ehrentraut S, Meyer C, Kaufmann M, Drexler HG, MacLeod RA (2015). Repressed BMP signaling reactivates NKL homeobox gene MSX1 in a T-ALL subset. Leuk Lymphoma.

[10] Jojic V, Shay T, Sylvia K, Zuk O, Sun X, Kang J, Regev A, Koller D, Best AJ, Knell J, Goldrath A, Joic V, Koller D, Immunological Genome Project Consortium (2013). Identification of transcriptional regulators in the mouse immune system. Nat Immunol.

[11] Odelberg SJ, Kollhoff A, Keating MT (2000). Dedifferentiation of mammalian myotubes induced by msx1. Cell.

[12] Rao J, Pfeiffer MJ, Frank S, Adachi K, Piccini I, Quaranta R, Araúzo-Bravo M, Schwarz J, Schade D, Leidel S, Schöler HR, Seebohm G, Greber B (2016). Stepwise Clearance of Repressive Roadblocks Drives Cardiac Induction in Human ESCs. Cell Stem Cell.

[13] Lee TI, Jenner RG, Boyer LA, Guenther MG, Levine SS, Kumar RM, Chevalier B, Johnstone SE, Cole MF, Isono K, Koseki H, Fuchikami T, Abe K (2006). Control of developmental regulators by Polycomb in human embryonic stem cells. Cell.

[14] Eskeland R, Leeb M, Grimes GR, Kress C, Boyle S, Sproul D, Gilbert N, Fan Y, Skoultchi AI, Wutz A, Bickmore WA (2010). Ring1B compacts chromatin structure and represses gene expression independent of histone ubiquitination. Mol Cell.

[15] Di Croce L, Helin K (2013). Transcriptional regulation by Polycomb group proteins. Nat Struct Mol Biol.

[16] Gao Z, Zhang J, Bonasio R, Strino F, Sawai A, Parisi F, Kluger Y, Reinberg D (2012). PCGF homologs, CBX proteins, and RYBP define functionally distinct PRC1 family complexes. Mol Cell.

[17] Junco SE, Wang R, Gaipa JC, Taylor AB, Schirf V, Gearhart MD, Bardwell VJ, Demeler B, Hart PJ, Kim CA (2013). Structure of the polycomb group protein PCGF1 in complex with BCOR reveals basis for binding selectivity of PCGF homologs. Structure.

[18] Nagel S, Venturini L, Marquez VE, Meyer C, Kaufmann M, Scherr M, MacLeod RA, Drexler HG (2010). Polycomb repressor complex 2 regulates HOXA9 and HOXA10, activating ID2 in NK/T-cell lines. Mol Cancer.

[19] Zhang J, Ding L, Holmfeldt L, Wu G, Heatley SL, Payne-Turner D, Easton J, Chen X, Wang J, Rusch M, Lu C, Chen SC, Wei L (2012). The genetic basis of early T-cell precursor acute lymphoblastic leukaemia. Nature.

[20] Ntziachristos P, Tsirigos A, Van Vlierberghe P, Nedjic J, Trimarchi T, Flaherty MS, Ferres-Marco D, da Ros V, Tang Z, Siegle J, Asp P, Hadler M, Rigo I (2012). Genetic inactivation of the polycomb repressive complex 2 in T cell acute lymphoblastic leukemia. Nat Med.

[21] Huether R, Dong L, Chen X, Wu G, Parker M, Wei L, Ma J, Edmonson MN, Hedlund EK, Rusch MC, Shurtleff SA, Mulder HL, Boggs K (2014). The landscape of somatic mutations in epigenetic regulators across 1,000 paediatric cancer genomes. Nat Commun.

[22] Fujikawa D, Nakagawa S, Hori M, Kurokawa N, Soejima A, Nakano K, Yamochi T, Nakashima M, Kobayashi S, Tanaka Y, Iwanaga M, Utsunomiya A, Uchimaru K (2016). Polycomb-dependent epigenetic landscape in adult T-cell leukemia. Blood.

[23] Swanson BJ, Jäck HM, Lyons GE (1998). Characterization of myocyte enhancer factor 2 (MEF2) expression in B and T cells: MEF2C is a B cell-restricted transcription factor in lymphocytes. Mol Immunol.

[24] Wilker PR, Kohyama M, Sandau MM, Albring JC, Nakagawa O, Schwarz JJ, Murphy KM (2008). Transcription factor Mef2c is required for B cell proliferation and survival after antigen receptor stimulation. Nat Immunol.

[25] Debnath I, Roundy KM, Pioli PD, Weis JJ, Weis JH (2013). Bone marrow-induced Mef2c deficiency delays B-cell development and alters the expression of key B-cell regulatory proteins. Int Immunol.

[26] Nagel S, Meyer C, Quentmeier H, Kaufmann M, Drexler HG, MacLeod RA (2008). MEF2C is activated by multiple mechanisms in a subset of T-acute lymphoblastic leukemia cell lines. Leukemia.

[27] Homminga I, Pieters R, Langerak AW, de Rooi JJ, Stubbs A, Verstegen M, Vuerhard M, Buijs-Gladdines J, Kooi C, Klous P, van Vlierberghe P, Ferrando AA, Cayuela JM (2011). Integrated transcript and genome analyses reveal NKX2-1 and MEF2C as potential oncogenes in T cell acute lymphoblastic leukemia. Cancer Cell.

[28] Nagel S, Venturini L, Meyer C, Kaufmann M, Scherr M, Drexler HG, Macleod RA (2011). Transcriptional deregulation of oncogenic myocyte enhancer factor 2C in T-cell acute lymphoblastic leukemia. Leuk Lymphoma.

[29] Oldridge DA, Wood AC, Weichert-Leahey N, Crimmins I, Sussman R, Winter C, McDaniel LD, Diamond M, Hart LS, Zhu S, Durbin AD, Abraham BJ, Anders L (2015). Genetic predisposition to neuroblastoma mediated by a LMO1 super-enhancer polymorphism. Nature.

[30] Sur IK, Hallikas O, Vähärautio A, Yan J, Turunen M, Enge M, Taipale M, Karhu A, Aaltonen LA, Taipale J (2012). Mice lacking a Myc enhancer that includes human SNP rs6983267 are resistant to intestinal tumors. Science.

[31] Pezzi N, Prieto I, Kremer L, Pérez Jurado LA, Valero C, Del Mazo J, Martínez-A C, Barbero JL (2000). STAG3, a novel gene encoding a protein involved in meiotic chromosome pairing and location of STAG3-related genes flanking the Williams-Beuren syndrome deletion. FASEB J.

[32] Sultana R, Yu CE, Yu J, Munson J, Chen D, Hua W, Estes A, Cortes F, de la Barra F, Yu D, Haider ST, Trask BJ, Green ED (2002). Identification of a novel gene on chromosome 7q11. 2 interrupted by a translocation breakpoint in a pair of autistic twins. Genomics.

[33] Oksenberg N, Ahituv N (2013). The role of AUTS2 in neurodevelopment and human evolution. Trends Genet.

[34] Kawamata N, Ogawa S, Zimmermann M, Niebuhr B, Stocking C, Sanada M, Hemminki K, Yamatomo G, Nannya Y, Koehler R, Flohr T, Miller CW, Harbott J (2008). Cloning of genes involved in chromosomal translocations by high-resolution single nucleotide polymorphism genomic microarray. Proc Natl Acad Sci U S A.

[35] Coyaud E, Struski S, Dastugue N, Brousset P, Broccardo C, Bradtke J (2010). PAX5-AUTS2 fusion resulting from t(7; 9)(q11.2; p13.2) can now be classified as recurrent in B cell acute lymphoblastic leukemia. Leuk Res.

[36] Denk D, Bradtke J, König M, Strehl S (2014). PAX5 fusion genes in t(7; 9)(q11 2; p13) leukemia: a case report and review of the literature. Mol Cytogenet.

[37] Zenatti PP, Ribeiro D, Li W, Zuurbier L, Silva MC, Paganin M, Tritapoe J, Hixon JA, Silveira AB, Cardoso BA, Sarmento LM, Correia N, Toribio ML (2011). Oncogenic IL7R gain-of-function mutations in childhood T-cell acute lymphoblastic leukemia. Nat Genet.

[38] Bedogni F, Hodge RD, Nelson BR, Frederick EA, Shiba N, Daza RA, Hevner RF (2010). Autism susceptibility candidate 2 (Auts2) encodes a nuclear protein expressed in developing brain regions implicated in autism neuropathology. Gene Expr Patterns.

[39] Gao Z, Lee P, Stafford JM, von Schimmelmann M, Schaefer A, Reinberg D (2014). An AUTS2-Polycomb complex activates gene expression in the CNS. Nature.

[40] Endoh M, Endo TA, Endoh T, Isono K, Sharif J, Ohara O, Toyoda T, Ito T, Eskeland R, Bickmore WA, Vidal M, Bernstein BE, Koseki H (2012). Histone H2A mono-ubiquitination is a crucial step to mediate PRC1-dependent repression of developmental genes to maintain ES cell identity. PLoS Genet.

[41] Cooper S, Dienstbier M, Hassan R, Schermelleh L, Sharif J, Blackledge NP, De Marco V, Elderkin S, Koseki H, Klose R, Heger A, Brockdorff N (2014). Targeting polycomb to pericentric heterochromatin in embryonic stem cells reveals a role for H2AK119u1 in PRC2 recruitment. Cell Rep.

[42] Tan J, Yang X, Zhuang L, Jiang X, Chen W, Lee PL, Karuturi RK, Tan PB, Liu ET, Yu Q (2007). Pharmacologic disruption of Polycomb-repressive complex 2-mediated gene repression selectively induces apoptosis in cancer cells. Genes Dev.

[43] Yu M, Mazor T, Huang H, Huang HT, Kathrein KL, Woo AJ, Chouinard CR, Labadorf A, Akie TE, Moran TB, Xie H, Zacharek S, Taniuchi I (2012). Direct recruitment of polycomb repressive complex 1 to chromatin by core binding transcription factors. Mol Cell.

[44] Winters T, McNicoll F, Jessberger R (2014). Meiotic cohesin STAG3 is required for chromosome axis formation and sister chromatid cohesion. EMBO J.

[45] Green RE, Krause J, Briggs AW, Maricic T, Stenzel U, Kircher M, Patterson N, Li H, Zhai W, Fritz MH, Hansen NF, Durand EY, Malaspinas AS (2010). A draft sequence of the Neandertal genome. Science.

[46] Li H, Radford JC, Ragusa MJ, Shea KL, McKercher SR, Zaremba JD, Soussou W, Nie Z, Kang YJ, Nakanishi N, Okamoto S, Roberts AJ, Schwarz JJ (2008). Transcription factor MEF2C influences neural stem/progenitor cell differentiation and maturation *in vivo*. Proc Natl Acad Sci U S A.

[47] Novara F, Beri S, Giorda R, Ortibus E, Nageshappa S, Darra F, Dalla Bernardina B, Zuffardi O, Van Esch H (2010). Refining the phenotype associated with MEF2C haploinsufficiency. Clin Genet.

[48] Sun T, Walsh CA (2006). Molecular approaches to brain asymmetry and handedness. Nat Rev Neurosci.

[49] Wang XW, Wei W, Wang WQ, Zhao XY, Guo H, Fang DC (2014). RING finger proteins are involved in the progression of barrett esophagus to esophageal adenocarcinoma: a preliminary study. Gut Liver.

[50] Ross K, Sedello AK, Todd GP, Paszkowski-Rogacz M, Bird AW, Ding L, Grinenko T, Behrens K, Hubner N, Mann M, Waskow C, Stocking C, Buchholz F (2012). Polycomb group ring finger 1 cooperates with Runx1 in regulating differentiation and self-renewal of hematopoietic cells. Blood.

[51] Pengelly AR, Kalb R, Finkl K, Müller J (2015). Transcriptional repression by PRC1 in the absence of H2A monoubiquitylation. Genes Dev.

[52] Jacques-Fricke BT, Gammill LS (2014). Neural crest specification and migration independently require NSD3-related lysine methyltransferase activity. Mol Biol Cell.

[53] Morishita M, Mevius D, di Luccio E (2014). *In vitro* histone lysine methylation by NSD1, NSD2/MMSET/WHSC1 and NSD3/WHSC1L. BMC Struct Biol.

[54] Mehra-Chaudhary R, Matsui H, Raghow R (2001). Msx3 protein recruits histone deacetylase to down-regulate the Msx1 promoter. Biochem J.

[55] Singh N, Gupta M, Trivedi CM, Singh MK, Li L, Epstein JA (2013). Murine craniofacial development requires Hdac3-mediated repression of Msx gene expression. Dev Biol.

[56] Wang J, Abate-Shen C (2012). The MSX1 homeoprotein recruits G9a methyltransferase to repressed target genes in myoblast cells. PLoS One.

[57] Wang J, Kumar RM, Biggs VJ, Lee H, Chen Y, Kagey MH, Young RA, Abate-Shen C (2011). The Msx1 Homeoprotein Recruits Polycomb to the Nuclear Periphery during Development. Dev Cell.

[58] Nagel S, Venturini L, Przybylski GK, Grabarczyk P, Meyer C, Kaufmann M, Battmer K, Schmidt CA, Drexler HG, Scherr M, Macleod RA (2009). NK-like homeodomain proteins activate NOTCH3-signaling in leukemic T-cells. BMC Cancer.

[59] Graux C, Cools J, Michaux L, Vandenberghe P, Hagemeijer A (2006). Cytogenetics and molecular genetics of T-cell acute lymphoblastic leukemia: from thymocyte to lymphoblast. Leukemia.

[60] Zuurbier L, Gutierrez A, Mullighan CG, Canté-Barrett K, Gevaert AO, de Rooi J, Li Y, Smits WK, Buijs-Gladdines JG, Sonneveld E, Look AT, Horstmann M, Pieters R (2014). Immature MEF2C-dysregulated T-cell leukemia patients have an early T-cell precursor acute lymphoblastic leukemia gene signature and typically have non-rearranged T-cell receptors. Haematologica.

[61] Jain N, Lamb AE, O‘Brien S, Ravandi F, Konopleva M, Jabbour E, Zuo Z, Jorgensen J, Lin P, Pierce S, Thomas D, Rytting M, Borthakur G (2016). Early T-cell precursor acute lymphoblastic leukemia/lymphoma (ETP-ALL/LBL) in adolescents and adults: a high-risk subtype. Blood.

[62] Huang da W, Sherman BT, Lempicki RA (2009). Systematic and integrative analysis of large gene lists using DAVID bioinformatics resources. Nat Protoc.

[63] Macleod RA, Kaufmann M, Drexler HG (2011). Cytogenetic analysis of cancer cell lines. Methods Mol Biol.

[64] Drexler HG (2010). Guide to leukemia-lymphoma cell lines.

[65] Kees UR, Ford J, Price PJ, Meyer BF, Herrmann RP (1987). PER-117: a new human ALL cell line with an immature thymic phenotype. Leuk Res.

[66] Scherr M, Battmer K, Ganser A, Eder M (2003). Modulation of gene expression by lentiviral-mediated delivery of small interfering RNA. Cell Cycle.

