# Supplementary material for: Deregulation of polycomb repressor complex 1 modifier AUTS2 in T-cell leukemia
Source: Oncotarget. 2016 Jun 13;7(29):45398–413. doi: 10.18632/oncotarget.9982 (PMC5216730; doi:10.18632/oncotarget.9982)
Supplement: Supplementary file 1 [file oncotarget-07-45398-s001.pdf]

Deregulation of polycomb repressor complex 1 modifier AUTS2 in T-cell leukemia

Supplementary Materials

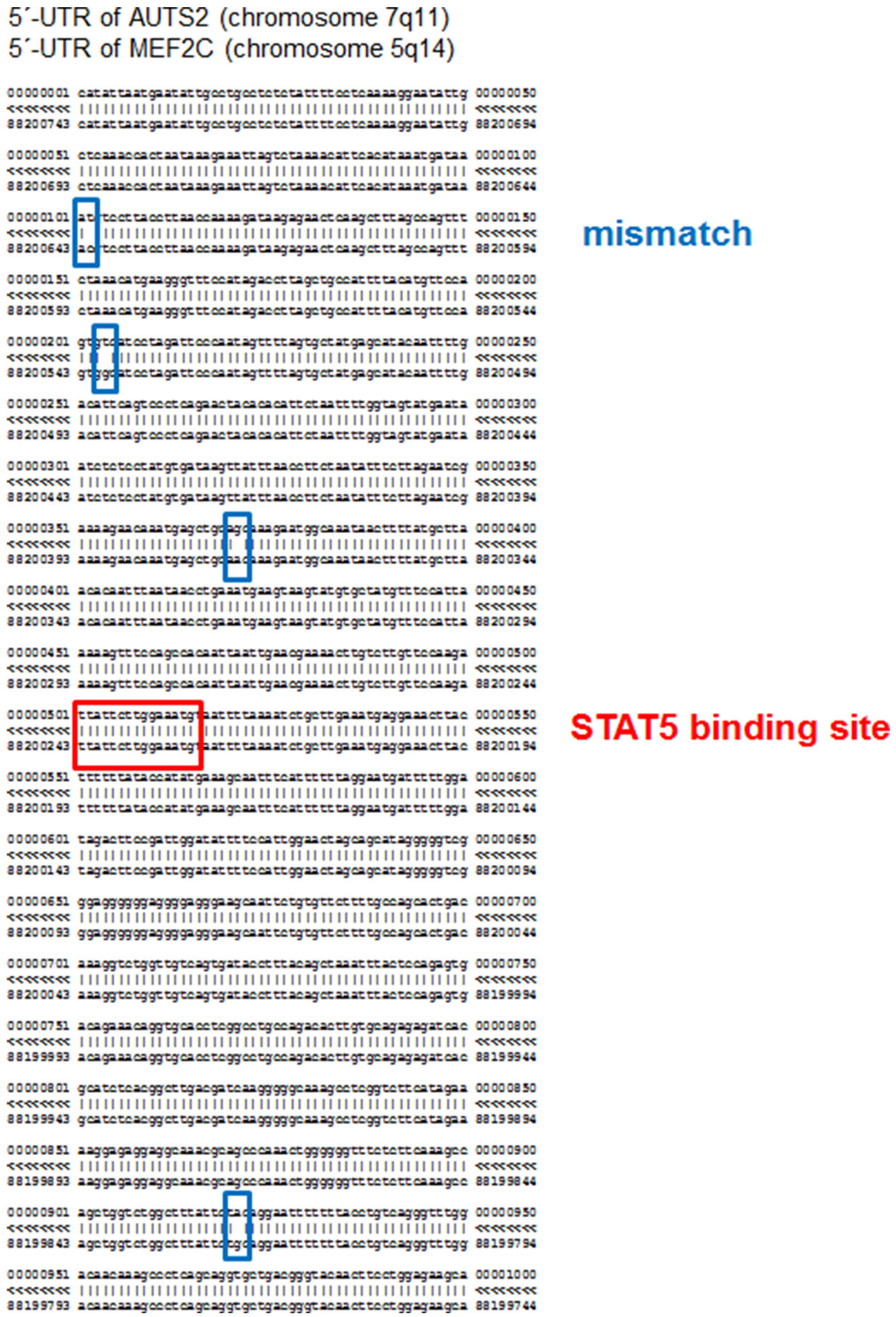

Supplementary Figure S1: Alignment of upstream regions of MEF2C and AUTS2. This alignment compares sequences (1000 bp) obtained from the upstream regions of AUTS2 (above) and of MEF2C (below). Mismatched positions are indicated in blue, the STAT5 binding site in red. Note the high conformity of both sequences including the STAT5 site.

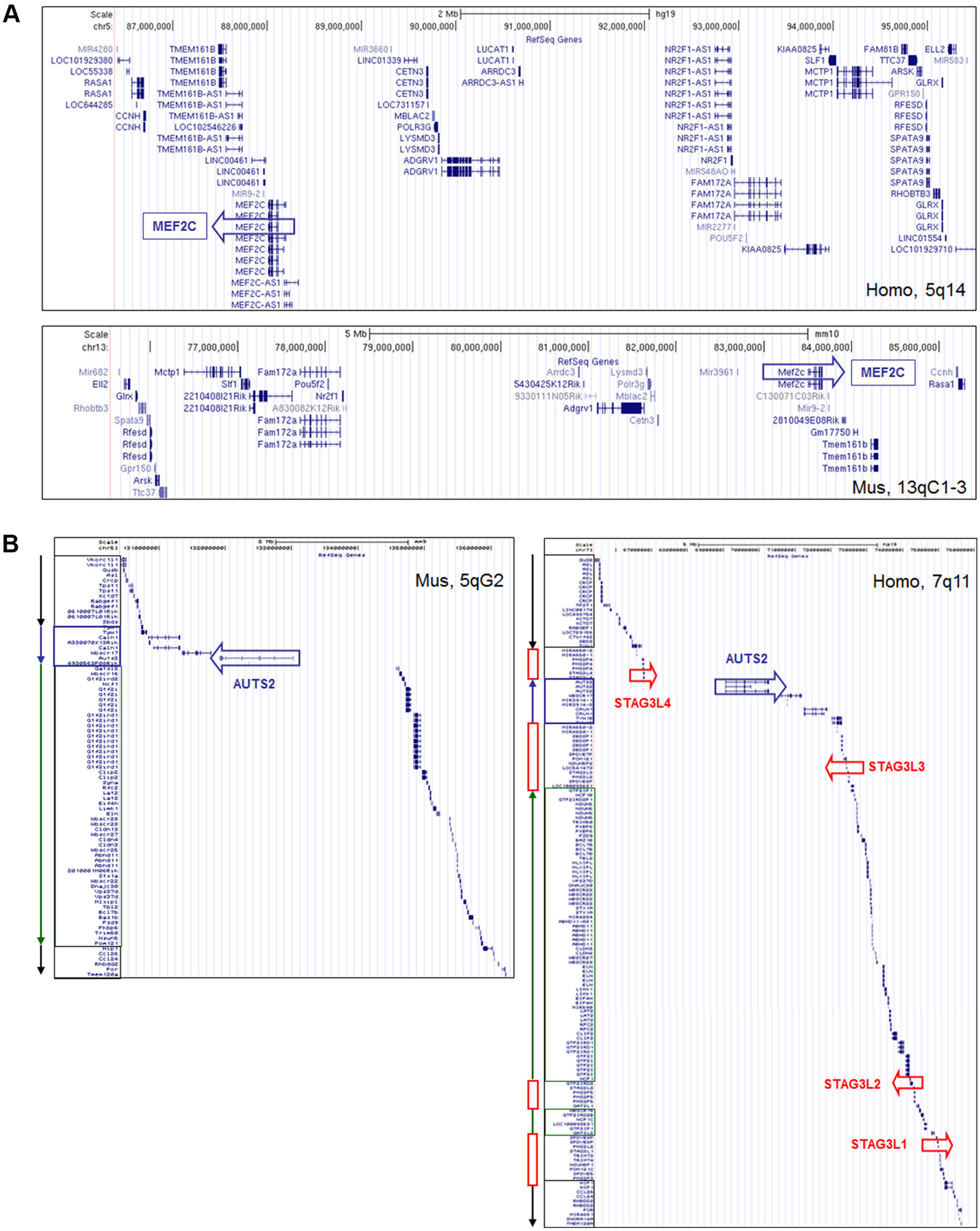

A
Scale
chr5:
hg19
2 Mb
RefSeq Genes
MEF2C
Homo, 5q14
Scale
chr13:
mm10
5 Mb
RefSeq Genes
MEF2C
Mus, 13qC1-3
B
Mus, 5qG2
AUTS2
Homo, 7q11
AUTS2
STAG3L4
STAG3L3
STAG3L2
STAG3L1

C

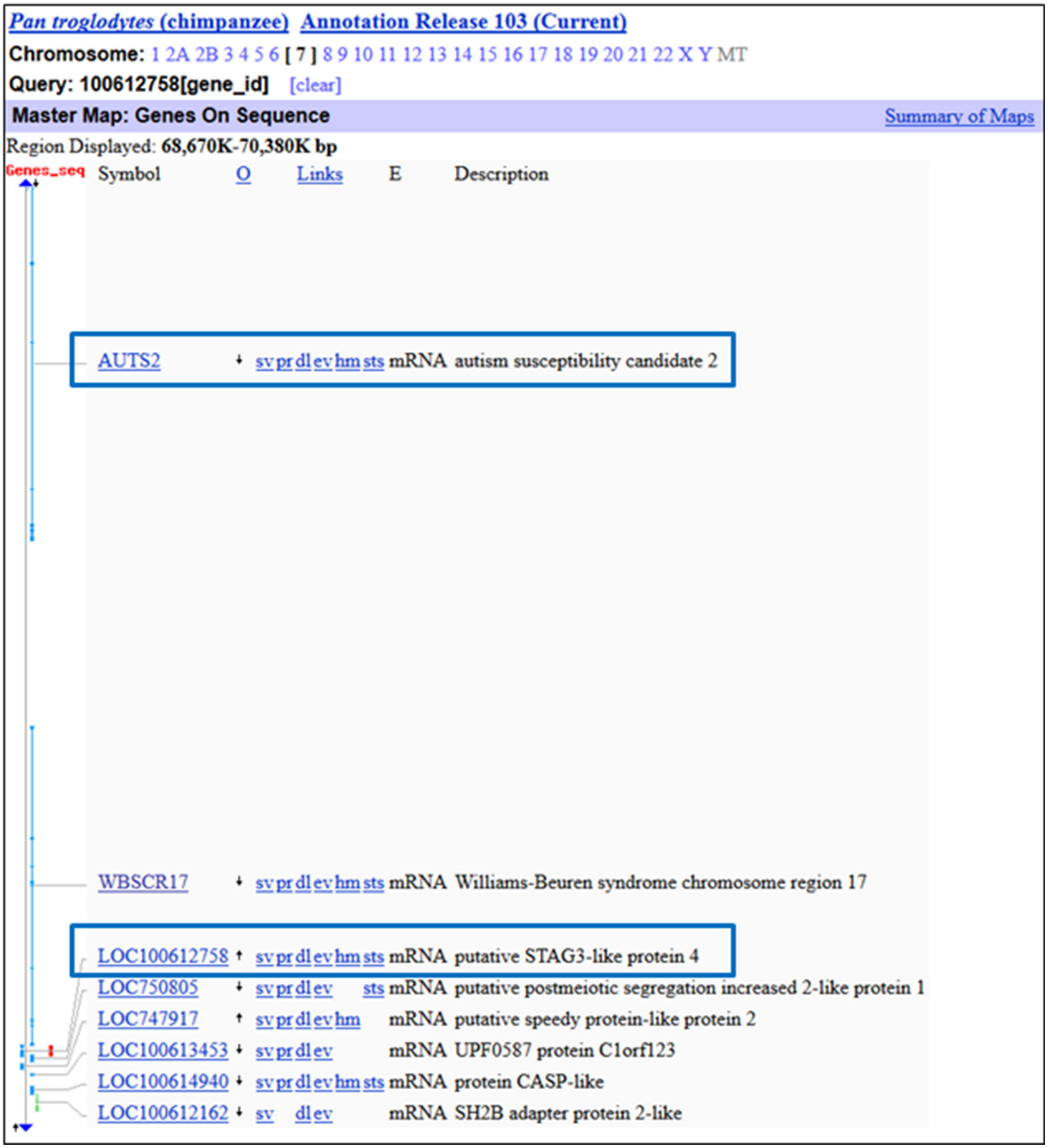

Supplementary Figure S2: AUTS2/STAG3L4 genomic region in mouse and chimp. (**A**) Genomic regions were obtained from the UCSC genome browser, containing MEF2C in human (above) and mouse (below). Please note the high conformity of both regions. (**B**) Genomic regions containing AUTS2 in mouse (left) and human (right). Note the differences between both regions (right). The mouse genome lacks STAG3-like genes. The indicated human region shows extended rearrangements of gene clusters as indicated by arrows and red boxes on the right side. (**C**) Genomic regions were obtained from the NCBI genome browser, showing gene arrangements of AUTS2, WBSCR17 and STAG3L4. Note the different arrangement as compared to the human genome.

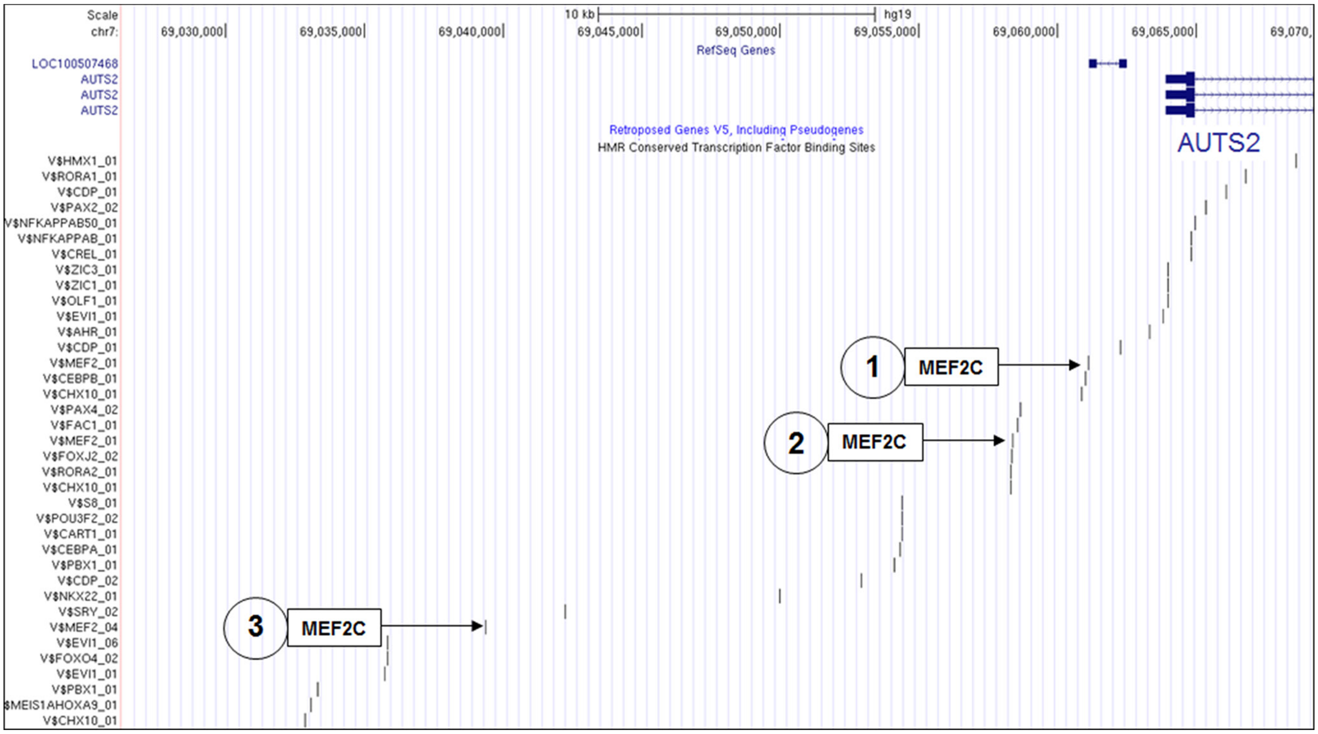

Supplementary Figure S3: MEF2C binding sites in the AUTS2 promoter region. The genomic region was obtained from the UCSC genome browser, showing the AUTS2 upstream region including potential TF binding sites. Note the presence of 3 potential sites for MEF2C.

```
gaagaaggaggaggaggaggaggagatgaggaggagaggaggaagaggag
gatgaggaggaggagaaaagaggaagaaaaagaggaagaggaagaagaag
aagaagaggaagaagaggaagatgctgttgttcgctgtcctggctgggct
accagcaaggtatttgccctacagccttgctcagcctcagcgagctgagc
ttcaaaattgggtcttaaggtcttctatccttgtaccctctcgggcaaac
ctccggaggccccggtgcaccgcgcgtccagccggcccaactcgagctag
aagccccaaccactgcccagtgcctgagttgcagtcttgggtcctttaga
aacctggagatgtgcgtaaaattcagatgccggtattcccgaacttcccc
aggcctcagcatatctcggcggcctgtggacagatgggaggctaccaatc  RUNX1-site (-1576 bp)
gctccggcgtccgcagcccgacccctgccgccagaccccggacgtcttcc
ggataataaagttcccgctctaattcattttccctaatctggacgcccct
aatctacagcttttattgcgcccagttaaaagtcgagggaattcgctgtc
cctccgcgctcggataattacccctaaatggccacggcagccccttgtgt
ttcctggagattagaaccccgcagtcatcaatggcagggccgagtgagcc
gccaatcacctccgctcactccctgagagccgctggcctgggccgcagga
ggagaggccataaagcgacaggcgcagaaaatggccaagccccgaccccg
cttcaggcagatcttgcatctcccctgaccccaactcatttttctgtct
ttattattattattattaacagcaaacattaaatcacgttttctccg
aaattggcaaaagaagagttctcaagtcccacactagagtccacctccag
gaactttcctcttttcccagcccccctcccccaaatgtgcctctcccctcc
aggatcagcggcccagagatagggacatgtcattgtttccgaggacgcgt
tgtttgaggtcaccttaaggccagatcggtgtttcattgacgattctgtt
ggttttatttcagtaagttctatatcaaaaggatctggcagctcctttcc
ccgcccgggcgctataggtgttgtgacccttgggacagaagttcagagcg
agggtgggggggggggaatacacatcgtgtatgaaaaccgactgcagatt
ctagataatcttacgtattctcacatccttggcactacaggaagctagct
tcttcccgcaaggtttactccagctctaagttagagacaaaggcccactt
ttacctcgaggtaaagtttacaagatttcagaacaggaagaaaatgaagg
tttggttttgtttcgtttcttgaaaagaagttaatagtatgtctttctcc
taggataaatagccatgcgtatttaaaaactatatataaaaggaatgtg
taagaaataacctcaactcaaattattgtggtagaagaagagggggggtc  RUNX1-site (-474 bp)
agacagtggagggggggcacagggaaacccagccacagactaaagagaaag
gtaaaagaagcagtagaggagagaaacaaggacggggaaaaaaagaggag
cggaaaagagggctgaggaggggaggggaggggaggagaggagggcagaa
gagaaggaacgagaacaagggaaaatcccccgggaacacagaaagataga
gacccaggggactcccgcagagagggcctcttgggcttcagcgcagagga
aagtttcccgggcacccccctctcctcccctgccctccgccgcctgggccc
tgccctgcgtgcccccaggcccagcgcgcctccgggcgagtccccaggag
cgcggcccaatggatcgctccgggcccgccccctcgcgcgctgattggcc
gccgccccgctggcctcgccttattagcaagttctctggggagccgcggt
AGGGCCCGGAGCCGGCGAGTGCTCCCGGGAACTCTGCCTGCGCGGCGGCA
GCGACCGGAGGCCAGGCCCAGCACGCCGGAGCTGGCCTGCTGGGGAGGGG
CGGGAGGCGCGCGCGGGAGGGTCCGCCCGGCCAGGGCCCCGGGCGCTCGC
AGAGGCCGGCCGCGCTCCCAGCCCGCCCGGAGCCCATGCCCGGCGGCTGG
CCAGTGCTGCGGCAGAAGGGGGGGCCCGGCTCTGCATGGCCCCGGCTGCT
GACATGACTTCTTTGCCACTCGGTGTCAAAGTGGAGGACTCCGCCTTCGG
CAAGCCGGCGGGGGGAGGCGCGGGCCAGGCCCCCAGCGCCGCCGCGGCCA
CGGCAGCCGCCATGGGCGCGGACGAGGAGGGGGCCAAGCCCAAAGTGTCC    MSX1 exon1
CCTTCGCTCCTGCCCTTCAGCGTGGAGGCGCTCATGGCCGACCACAGGAA
GCCGGGGGCCAAGGAGAGCGCCCTGGCGCCCTCCGAGGGCGTGCAGGCGG
CGGGTGGCTCGGCGCAGCCACTGGGCGTCCCGCCGGGGTCGCTGGGAGCC
CCGGACGCGCCCTCTTCGCCGCGGCCGCTCGGCCATTTCTCGGTGGGGGG
ACTCCTCAAGCTGCCAGAAGATGCGCTCGTCAAAGCCGAGAGCCCCGAGA
AGCCCGAGAGGACCCCGTGGATGCAGAGCCCCCGCTTCTCCCCGCCGCCG
GCCAgtgagtagccagaacccaggcgcagagggagggggccgggtggggg
```

Supplementary Figure S4: RUNX1 binding sites in the MSX1 promoter region. The genomic sequence was obtained from the UCSC genome browser, showing the MSX1 upstream region including two potential binding sites for RUNX1.

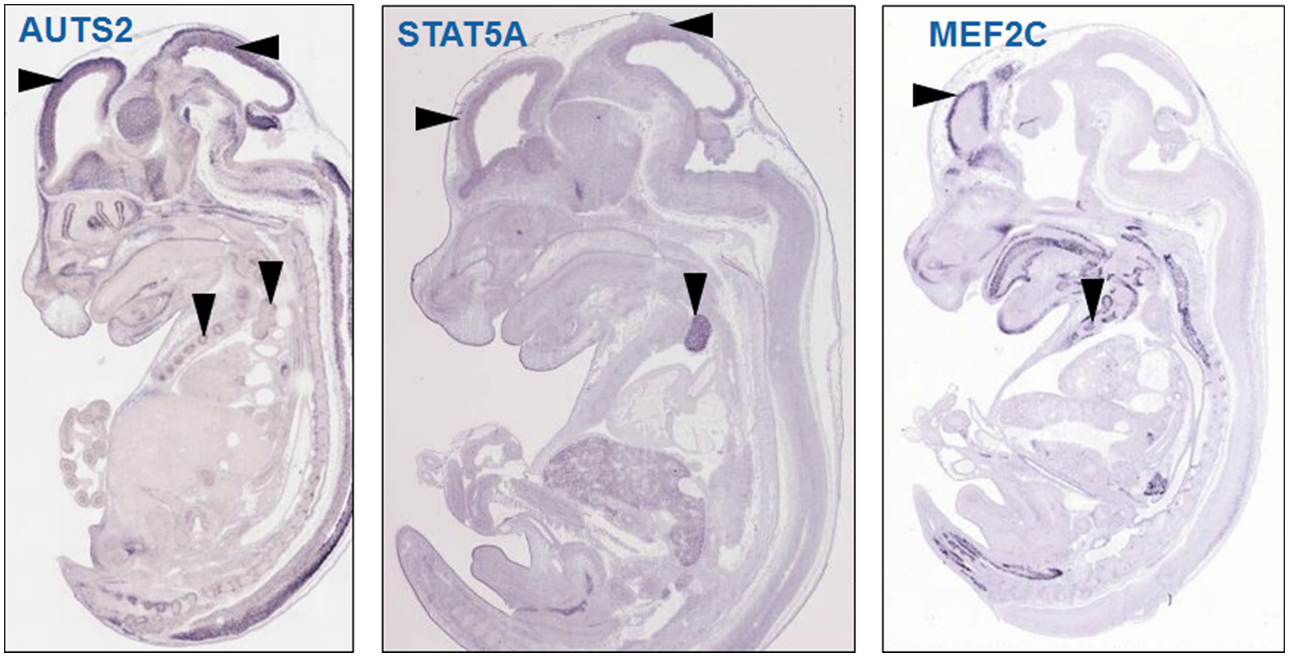

Supplementary Figure S5: AUTS2, MEF2C, STAT5 expression in mouse embryos. Immuno-staining data of mouse embryos for AUTS2 (left), STAT5A (middle), and MEF2C (right) were obtained from eurexpress.org. Triangles indicate selected embryonal regions with positive signals: brain, rips (bone marrow), thymus.

Supplementary Table S1: Expression profiling data of 5 T–ALL cell lines. See Supplementary_Table_S1
